# A review of the benefits and risks of nonsteroidal anti-inflammatory drugs in the management of mild-to-moderate osteoarthritis

**DOI:** 10.1186/1750-4732-3-1

**Published:** 2009-01-06

**Authors:** A Mark Fendrick, Bruce P Greenberg

**Affiliations:** 1 Internal Medicine/Health Management and Policy, University of Michigan Medical Center, 300 North Ingalls Building, Room 7E06, Ann Arbor, MI 48109-0429, USA; 2 BlueSpark Healthcare Communications, 150 Allen Road, Suite 202, Basking Ridge, NJ 07920, USA

## Abstract

This review is intended to provide physicians with an overview of the benefits and risks associated with the use of nonsteroidal anti-inflammatory drugs (NSAIDs) in the management of their patients with mild-to-moderate osteoarthritis (OA). New information on the inflammatory component of OA and the cardiovascular (CV) risk associated with cyclooxygenase (COX)-2-specific inhibitors has prompted efforts to revise the current recommendations for the use of NSAIDs in the treatment of patients with OA. Clinical studies have shown that naproxen and ibuprofen are significantly more effective at reducing OA pain than is acetaminophen, the traditional first-line therapy, which has no apparent anti-inflammatory activity in the joints. The theoretical advantage of COX-2-specific inhibitors in reducing gastrointestinal (GI) toxicity has been demonstrated by clinical studies. GI complications can be reduced by using lower NSAID doses for the shortest duration or with a concomitant proton-pump inhibitor. All prescription NSAIDs carry a black box warning regarding CV risks; these risks vary among the NSAIDs. While ibuprofen and diclofenac are associated with an increased CV risk, naproxen was associated with a neutral CV risk relative to placebo. Ibuprofen, but not naproxen, attenuates the antiplatelet effects of aspirin. An understanding of the risks and benefits is important when choosing an NSAID. An exhaustive search of the medical literature since 1990 was conducted using the words "ibuprofen," "naproxen," "COX-2-specific NSAIDs," "nonspecific NSAIDs," "low-dose aspirin," and "nonprescription dosage." Databases searched included MEDLINE, EMBASE, and SCISEARCH. This article provides primary care physicians with the information needed to assist them in making more informed decisions in managing patients experiencing mild-to-moderate OA pain.

## Introduction

Patients with musculoskeletal diseases such as osteoarthritis (OA) are typically managed with a combination of nonpharmacologic modalities and pharmacologic agents that are chosen to pose a minimal risk of side effects. The main goals of this approach are to control pain and improve function and health-related quality of life. Nonsteroidal anti-inflammatory drugs (NSAIDs) are the most frequently prescribed drugs for managing musculoskeletal pain. The clinical usefulness of these agents in relieving mild-to-moderate pain needs to be balanced with a consideration of adverse effects, including gastrointestinal (GI) bleeding and perforation[1]. Therefore, clinicians treating patients with OA need to be aware of the safety and efficacy profiles of currently available prescription and over-the-counter (OTC) NSAIDs. A systemic search of the medical literature for the treatment of mild-to-moderate OA was conducted between January 1990 and February 2008. Key search terms included: "ibuprofen," "naproxen," "NSAIDs," "COX-2-specific NSAIDs," "nonspecific NSAIDs," "low-dose aspirin," and "nonprescription dosage." Randomized clinical trials, epidemiologic or observational studies, meta-analyses and systemic reviews, and cardiovascular and GI risk were also reviewed. Databases searched included MEDLINE, EMBASE, and SCISEARCH. This review of the NSAID class will help primary care physicians choose the appropriate treatment for their patients with OA and musculoskeletal pain.

### NSAIDs: overview and mechanism of action

The NSAIDs are a heterogeneous group of compounds that exhibit anti-inflammatory, analgesic, and antipyretic properties. NSAIDs that have been approved by the US Food and Drug Administration (FDA) for OTC analgesic use can be separated into three groups: salicylates, represented by aspirin; propionic acid derivatives, including ibuprofen and naproxen sodium; and the para-aminophenols, represented by acetaminophen (Table 1). While ibuprofen and naproxen are both considered traditional NSAIDs, acetaminophen is not. Although acetaminophen has weak cyclooxygenase (COX) inhibition activity, it appears to have little anti-inflammatory activity, especially in the high-peroxide environment of OA-affected joints[2].

**Table 1:** Commercially available nonprescription NSAIDs

Generic drug name	Principal brand name in United States	OTC dosage	Maximum OTC daily dosage^a^
Salicylates			
Aspirin	Anacin^®^, Bayer aspirin^®^, Ecotrin^®^, Bufferin^®^, St Joseph^® ^aspirin	650–1000 mg q4-6h	4000 mg
Propionic acid derivatives			
Ibuprofen	Advil^®^, Midol^®^, Motrin^®^, Nuprin^®^	200–400 mg q4-6h	1200 mg^b^
Naproxen	Aleve^®^	220 mg q8-12h^b^	660 mg^b^
Para-aminophenols			
Acetaminophen	Tylenol^® ^(plus many combination products)	650–1000 mg q4-6h	4000 mg^b^

NSAIDs = nonsteroidal anti-inflammatory drugs; OTC = over-the-counter.

^a^Administered over a 10-day period.

^b^Persons aged ≥60 years should check with a physician prior to taking any medication.

The inhibition of prostaglandin production by NSAIDs was first demonstrated in 1971; this work ultimately led to the researchers receiving a Nobel Prize in Medicine[3]. This activity, distinct from that of other analgesics, was speculated to be the action that mediated the gastric side effects commonly observed with the NSAIDs[3]. The mechanism of action of the NSAIDs is based on the inhibition of the COX isoenzymes, COX-1 and COX-2. Nonselective NSAIDs inhibit both COX-1 and COX-2, whereas COX-2-specific inhibitors have a minimal effect on COX-1. COX-1 is present constitutively in most normal cells and tissues; it stimulates prostaglandin synthesis, regulates platelet aggregation, and modulates vascular homeostasis, the mucosal integrity of the GI tract, and the functioning of the renal system. COX-2, in contrast, is a key mediator of inflammation; it is induced in response to inflammatory stimuli, initiating pain and the inflammatory response[4].

The analgesic effects of NSAIDs have been attributed to the inhibition of COX-2, while the GI side effects are thought to be derived from the inhibition of COX-1. COX-2-specific inhibition was anticipated to preserve effective anti-inflammatory activity while causing little or no associated GI toxicity[5,6]. However, the inhibition of COX-2 results in reduced production of prostaglandin I_2 _by vascular endothelium, which may contribute to an increased risk for thrombosis[7]. In addition, antiplatelet effects of NSAIDs are thought to be derived primarily from COX-1 inhibition; thus, the diminished antiplatelet effect is due to the imbalance between prostacyclin and thromboxane production[5,7].

### NSAID benefits in pain management

In acute settings, the analgesic effects of non-aspirin NSAIDs are comparable to those of starting doses of opioids[5,8]. However, unlike opioids, NSAIDs have a ceiling dose above which no additional pain relief occurs, although higher doses do mediate anti-inflammatory effects[8]. Clinical studies in patients with arthritis have shown that non-aspirin NSAIDs are comparable to aspirin in reducing pain, joint swelling, and duration of morning stiffness, as well as in improving strength and mobility[9]. In randomized, double-blind, controlled trials using acetaminophen as the comparator, diclofenac, a nonselective NSAID[10], and celecoxib, a COX-2-specific NSAID[11], demonstrated greater relief of pain and stiffness.

A growing body of evidence has identified inflammation as an important component of the pathogenesis of OA and OA-related pain[12,13]. Inflammatory cytokines and proteases contribute to a varying extent in the process of joint destruction[12] and exacerbation of nociception[14]. Individual differences in both the degree of inflammation and susceptibility to nociception sensitization may explain, at least in part, why some patients with limited disease radiographically have severe pain, while other patients with more severe deterioration of the joint have minimal pain[15]. For this reason, the use of analgesics without anti-inflammatory properties in the joints, such as acetaminophen, would only provide pain relief without clinically beneficial anti-inflammatory activity[16,17].

Among the nonselective NSAIDs, both ibuprofen and naproxen are available in OTC and prescription dosing options. The safety and efficacy of OTC dosing of ibuprofen and naproxen have been assessed in large clinical trials[18]. OTC doses of both ibuprofen and naproxen provided significantly improved pain control over placebo. Similarly, ibuprofen[19] and naproxen[20] have been shown to be significantly more effective than acetaminophen for the treatment of pain associated with OA. A comparison of ibuprofen with acetaminophen found that pain relief was superior with ibuprofen, both after single dosing (ibuprofen 400 mg, acetaminophen 1000 mg) and after 14 days of treatment (ibuprofen 400 mg, acetaminophen 1000 mg)[19]. Similarly, pain relief with naproxen versus placebo at dosages of 660 mg/day (440 mg/day in patients ≥65 years old) was greater than that of acetaminophen versus placebo at dosages of 4000 mg/day[20].

Patients without contraindicating conditions (pain persisting for >2 weeks, pain with nausea or severe vomiting, weakness in any limb, pain in a red, hot, or swollen joint, suspected fracture, pelvic or abdominal pain, suspected pregnancy, and increased intensity of pain or any change in the character of pain), can self-treat using OTC NSAIDs[21] over a period of 10 days or less. Use of these agents for more than 10 days, even at recommended nonprescription doses, requires the supervision of a physician, as long-term self-treatment could mask a more serious condition requiring a physician's intervention[22].

### NSAID risks

#### GI effects

NSAID use has long been associated with GI side effects, including dyspepsia, heartburn, and nausea. Serious clinical GI complications occur in up to 4% of patients taking nonselective NSAIDs at prescription dosages[23]. Endoscopic studies suggest that up to 30% of regular nonselective NSAID users may develop gastric or duodenal ulcers[24]. The risk for developing GI complications is greater in patients who are treated with high doses of NSAIDs or treated for extended periods of time, and also in patients who take multiple NSAIDs concomitantly. Patients with prior ulcer or bleeding complications, patients ≥60 years of age, and patients taking concomitant aspirin, corticosteroids, or anticoagulants are also at increased risk[25]. NSAID-related GI complications and mortality have declined since 1992 in response to the use of lower NSAID doses and the use of combination therapy with proton-pump inhibitors[26].

The COX-2-specific inhibitors were expected to deliver equivalent pain relief to that of the nonselective NSAIDs but without the accompanying risk of GI complications. Although they did not eliminate this risk altogether, most clinical trials involving COX-2-specific inhibitors did show a lower incidence of upper GI adverse effects than the nonselective inhibitors[24,27]. However, the Celecoxib Long-term Arthritis Safety Study (CLASS), which combined the results from two prospective trials comparing celecoxib with diclofenac and ibuprofen, showed no significant difference between the groups in the rate of upper GI ulcer complications (0.7% for patients receiving celecoxib versus 1.5% for patients receiving either of the nonselective NSAIDs)[27-29]. The primary end point measured was the presence of complicated ulcers (i.e. ulcers requiring hospitalization and resulting in significant morbidity, mortality, or both). When patients receiving concomitant low-dose aspirin (about 20% of the total) were excluded from the analysis, celecoxib was associated with a significantly lower incidence of symptomatic ulcers and/or ulcer complications compared with the nonselective NSAIDs[29,30].

#### Cardiovascular effects

A number of large clinical trials have revealed that the COX-2-specific inhibitors are associated with an increased risk of cardiovascular (CV) toxicity[24,31,32]. The Vioxx Gastrointestinal Outcomes Research (VIGOR) study demonstrated a 5-fold increase in the incidence of myocardial infarction in rheumatoid arthritis patients treated with the COX-2-specific inhibitor rofecoxib, as compared with naproxen[24]. In a randomized, placebo-controlled clinical trial evaluating the COX-2 inhibitor rofecoxib for the chemoprevention of colorectal neoplasia (The Adenomatous Polyp Prevention On Vioxx [APPROVe] trial), the patients randomized to rofecoxib had a 2-fold increase in vascular events (46 thrombotic events) compared with patients who received placebo (26 events)[31]. This trial involved 2586 patients who had a history of colorectal adenomas and were treated for 18 months. These data led the manufacturer to voluntarily withdraw the product from the market in 2004[33]. The fact that an increased risk of serious CV events was also seen in clinical trials with the COX-2-specific inhibitors parecoxib and valdecoxib suggested a possible class effect[34].

Currently, the only COX-2-specific NSAID available in the United States is celecoxib, and the evidence for increased CV risk with this drug is conflicting. The Adenoma Prevention with Celecoxib (APC) study compared 2 dosages of celecoxib (200 mg twice daily and 400 mg twice daily) with placebo in 2035 patients[32]. Results showed a 2- to 3-fold increase in relative risk of serious CV events with celecoxib versus placebo after a mean duration of 33 months. The Prevention of Colorectal Sporadic Adenomatous Polyps (PreSAP) trial, in contrast, showed no increased CV risk with celecoxib compared with placebo in 1561 patients[35]. The difference in celecoxib dosing between the two trials – 400 mg once daily in PreSAP, compared with 200 mg or 400 mg twice daily in APC – suggests that the occurrence of CV events may be related to both the dose and the pharmacokinetics of celecoxib[35].

Because of the CV risks of COX-2-specific NSAIDs, and despite the lack of a definitive conclusion about the potential CV risk of nonselective NSAIDs, the FDA requires a "black box" warning on all prescription NSAIDs, stating that "NSAIDs may cause an increased risk of serious CV thrombotic events, myocardial infarction, and stroke."[23] This issue, and related issues on the comparative efficacy and GI safety of the NSAIDs, was sufficiently important to prompt the Agency for Healthcare Research and Quality to commission a thorough assessment of all the available evidence on NSAIDs to best answer these questions[30]. This systematic review of the evidence showed that the CV safety of naproxen was moderately superior to that of any COX-2-specific NSAID. Data showed 3.3 additional myocardial infarctions for every 1000 patients treated with any COX-2 inhibitor instead of naproxen for 1 year. In contrast, the CV safety of nonselective NSAIDs other than naproxen (data primarily on diclofenac and ibuprofen) was found to be comparable to that of the COX-2-specific NSAIDs[30]. In addition, observational studies and indirect analyses of randomized trials indicated that the CV risk of naproxen was neutral relative to placebo, whereas comparisons of ibuprofen and diclofenac with placebo showed an increased risk of CV events[30,36].

#### Effects on concomitant use of low-dose aspirin

Aspirin is widely used by patients with OA as prophylaxis against CV disease. Drug interactions between aspirin and analgesics commonly used by these patients are therefore important to recognize. In particular, ibuprofen appears to antagonize the platelet-inhibiting activity of aspirin, limiting its cardioprotective effects[37]. This antagonism may help to explain why there were a higher number of CV events in patients taking ibuprofen plus low-dose aspirin than in patients taking lumiracoxib plus aspirin in a post-hoc analysis of CV safety data from the Therapeutic Arthritis Research and Gastrointestinal Event Trial (TARGET)[38]. Ibuprofen's interference with the cardioprotective effect of low-dose aspirin is thought to be mediated by the competitive inhibition of COX-1[39]. It is not known if this is the case for other nonselective NSAIDs. The FDA has approved enhanced warning labels for all OTC NSAIDs. These new labels will provide consumers with more information about potential CV, GI, and allergic risks[40].

In contrast, when naproxen is coadministered with low-dose aspirin, its antiplatelet effects (as measured by serum thromboxane inhibition) are equivalent to those of low-dose aspirin alone. During the dosing period, this is true for both prescription doses[41] and nonprescription doses[42] of naproxen. When measured independently, the antiplatelet effects of low-dose aspirin, OTC-dose naproxen (220 mg bid and tid), and prescription-dose naproxen (550 mg bid) are equivalent[43].

COX-2-specific inhibitors seem to lose their favorable GI safety profile when they are given concomitantly with low-dose aspirin[30,44]. Data from the CLASS study showed that celecoxib was associated with a significantly lower rate of serious GI adverse events than were nonselective NSAIDs among the 80% of patients not receiving low-dose aspirin, but not among the 20% of patients receiving low-dose aspirin[29]. Similar findings were reported in TARGET, which compared the COX-2-specific NSAID lumiracoxib with naproxen and ibuprofen in patients with OA[27].

### Choosing an NSAID for an OA patient

Various clinical guidelines have been proposed for the management of patients with OA[45-47], the most recent of which was published in February 2008[44]. The 1995 American College of Rheumatology (ACR) guidelines for the medical management of OA of the hip recommended acetaminophen as first-line therapy, permitting the use of a nonselective NSAID if pain control with acetaminophen was inadequate. This recommendation was based on the association of nonselective NSAIDs with GI toxicity[46]. The updated 2000 ACR guidelines recommended COX-2-specific inhibitors as second-line treatment choices after acetaminophen[45]. This recommendation was based on the improved GI safety of these agents over that of nonselective NSAIDs, especially in high-risk populations. However, rofecoxib, one of the COX-2 specific inhibitors, was voluntary withdrawn from the US market because of an increased risk of serious CV events[33]. The ACR guidelines also recommended use of a nonselective NSAID instead of acetaminophen, or with acetaminophen as add-on therapy, if pain control was insufficient[45]. Similarly, the American Pain Society guidelines from 2002 also recommended acetaminophen as a first-line agent[47].

The Osteoarthritis Research Society International (OARSI) has recently published (2008) its recommendations for the management of OA of the knee and hip[44]. These recommendations are based on a comprehensive review of the published literature and the existing guidelines for the treatment of OA. Consistent with existing guidelines, OARSI indicates that acetaminophen (up to 4 g/day) can be used as initial therapy in patients with mild-to-moderate pain from OA of the knee and hip. If response to acetaminophen is inadequate, or if pain or inflammation (or both) is severe, OARSI recommends that alternative pharmacotherapy be considered. In patients with symptomatic OA of the knee or hip, OARSI recommends use of the lowest effective dose of NSAIDs, for the shortest possible period of time. The important point to note in these 2008 guidelines is that acetaminophen is used for the treatment of mild-to-moderate pain, while NSAIDs are recommended in symptomatic patients who have both pain and inflammation. These updated recommendations are based on previously cited data[16,17] showing that analgesics such as acetaminophen relieve pain but do not provide anti-inflammatory activity within the joint. Patients at increased GI risk should receive either a proton-pump inhibitor or misoprostol concomitantly with their NSAID for gastroprotection.

In light of the increased risk for GI and CV events in patients with OA, a simple stratification grid was recently proposed to guide clinicians in selecting the most appropriate NSAID for OA patients by evaluating their CV and GI risk factors (Table 2)[48]. The stratification grid directs clinicians to choose monotherapy with a nonselective NSAID for patients who do not have CV risk factors and are at low risk for GI events. For patients without CV risk but with elevated risk for GI complications and who are not taking aspirin, the recommended treatment is monotherapy with a COX-2-specific inhibitor or combination therapy with a nonselective NSAID plus a proton-pump inhibitor to mitigate GI risk[49,50]. Patients who are at increased CV risk and who require aspirin prophylaxis should be treated with a nonselective NSAID that has been proven to not compromise the antiplatelet effects of aspirin. Although aspirin is approved by the FDA for primary and secondary prevention in a number of CV patient populations[51], concomitant administration of aspirin with ibuprofen has been shown to decrease the cardioprotective effects of aspirin[37]. This effect has not been seen in patients treated concomitantly with aspirin and naproxen[38]. If these patients require gastroprotection, a proton-pump inhibitor is again suggested; COX-2-specific inhibitors should be avoided in these patients[25,48].

**Table 2:** NSAID selection stratification grid

	No or low NSAID GI risk	NSAID GI risk
No CV risk (without aspirin)	• Nonselective NSAID (cost consideration)	• COX-2-specific inhibitor or nonselective NSAID + proton-pump inhibitor^a^
		• COX-2-specific inhibitor + proton-pump inhibitor for patients with prior GI bleeding

Yes CV risk (with aspirin)	• Naproxen^b^	• Proton-pump inhibitor, irrespective of NSAID
	• Addition of proton-pump inhibitor if GI risk of aspirin/NSAID combination warrants gastroprotection	• Naproxen if CV risk outweighs GI risk
		• COX-2-specific inhibitor + proton-pump inhibitor for patients with previous GI bleeding

COX-2 = cyclooxygenase-2; CV = cardiovascular; GI = gastrointestinal; NSAID = nonsteroidal anti-inflammatory drug.

^a^Misoprostol at full dosage (200 μg qid) may be substituted for a proton-pump inhibitor.

^b^Nonselective or selective (low-dose) inhibitor without established aspirin interaction if naproxen is ineffective.

From Scheiman and Fendrick ^48^

## Conclusion

The management of patients with mild-to-moderate OA involves careful consideration of the benefits and risks of currently available NSAIDs. Medication should be selected on the basis of pain intensity, inflammation, and GI and CV risk factors. Recent data seems to indicate that acetaminophen may not be as effective as the NSAIDs in symptomatic patients with pain and inflammation.

## Competing interests

A. Mark Fendrick receives research funding and/or consulting fees from Pfizer Inc, Merck & Co., Inc., Bayer HealthCare, AstraZeneca Pharmaceuticals LP, and the Perrigo Company. Bruce P. Greenberg holds stock in Wyeth Pharmaceuticals.

## Authors' contributions

AMF and BPG were involved in the conception, drafting, revising, and final approval of the important intellectual content for this manuscript.
